# Unveiling hidden sources of noise

**DOI:** 10.7554/eLife.102878

**Published:** 2024-10-01

**Authors:** Morgan Fitzgerald, Eena Kosik, Bradley Voytek

**Affiliations:** 1 Neurosciences Graduate Program, University of California, San Diego La Jolla United States; 2 Department of Cognitive Science, University of California, San Diego La Jolla United States; 3 Halıcıoğlu Data Science Institute, University of California, San Diego La Jolla United States; 4 Kavli Institute for Brain and Mind, University of California, San Diego La Jolla United States

**Keywords:** neuroscience, aging, aperiodic activity, cardiac activity, neural 1/f dynamics, Human

## Abstract

Changes in neural activity thought to reflect brain aging may be partly influenced by age-dependent signals ‘leaking’ from the heart.

**Related research article** Schmidt F, Danböck SK, Trinka E, Klein DP, Demarchi G, Weisz N. 2024. Age-related changes in 'cortical' 1/f dynamics are explained by cardiac activity. *eLife* **13**:RP100605. doi: 10.7554/eLife.100605.

In March 2014, a team of cosmologists made headlines around the world when they reported possible evidence for cosmic inflation – an extremely short period during which the universe rapidly expanded immediately after the Big Bang (Ade et al., 2014). However, it later emerged that some of the signal this result was based on had been produced by something seemingly quite insignificant: cosmic dust (Cowen, 2015). This episode underscored how small, apparently innocuous factors can have colossal implications when studying the universe. The same is true in neuroscience, with perhaps even higher stakes.

We are all impacted by changes in our brains, especially in the latter part of our lives (Alzheimer’s Association, 2019). As the global population ages at an unprecedented rate, neuroscientists are leveraging a vast array of tools to confront the complexities of brain aging at every biological scale. When recording brain activity, these researchers must exhibit the same precision demanded of cosmologists, carefully accounting for the various sources of non-neural noise stemming from their instruments, from their lab environment, or from biological signals such as eye movements or skin conductance. Now, in eLife, Fabian Schmidt and colleagues at Paris Lodron University of Salzburg and other institutions in Austria report that cardiac signals may be contaminating recordings thought to be of neural origin (Schmidt et al., 2024). Just as space dust misled cosmologists, electrical activity from the heart might distort our understanding of brain aging by influencing what is thought to be purely ‘neural’ data.

To understand these results, it is important to know that brain activity is composed of electrical signals that are either periodic (rhythmic) or aperiodic (non-rhythmic). Periodic or ‘neural’ oscillations occur at regular intervals, showing up as consistent patterns or waves. Extensively studied over the past century, these are linked to cognitive processes, perception, and various disease states (Buzsáki and Draguhn, 2004). Aperiodic activity, in contrast, does not follow a predictable pattern. Historically, it has largely been treated as noise to be averaged away in analyses, but mounting evidence suggests it may in fact be a signal in its own right. Crucially, previous studies have shown that neural aperiodic activity changes with age (Donoghue et al., 2020). However, cardiac signals contain both periodic and aperiodic components as well.

The work by Schmidt et al., which relied on datasets from various institutions, involved examining electrocardiograms (ECG) as well as neural data from electroencephalography (EEG) and resting state magnetoencephalography (MEG) – 1104 and 1282 recordings, respectively – obtained from individuals aged 18–92. The team extracted the aperiodic signal from both the neural and cardiac datasets and explored how some of its features evolved with age.

Their analyses revealed that cardiac artifacts significantly influenced the age-related changes in aperiodic activity detected in brain recordings. In fact, certain MEG and EEG changes could be attributed to the aging of the heart rather than of the brain, as had previously been thought.

Schmidt et al. then showed that attempting to ‘clean’ this cardiac contamination using a standard method called independent component analysis was insufficient. Despite the approach attempting to separate cardiac contributions from the neural data, residual aperiodic signals from the heart could still be detected in MEG and EEG recordings thought to capture only brain activity.

Moreover, results across different datasets pointed to a complex pattern of age-related changes in aperiodic neural activity. Researchers often examine how much of the electrical activity (or ‘power’) from the brain or heart is concentrated across various frequencies – with the slope of the resulting curve, which is how aperiodic activity is often measured, usually changing with age. In the brain, this curve is steeper in youth (as lower frequencies concentrate more power) and flattens in old age, possibly due to an increase in neural noise (Voytek et al., 2015; Figure 1). This effect could potentially be driven by the heart, as Schmidt et al. found a similar age-dependent flattening pattern in the cardiac aperiodic activity leaking into brain recordings. However, an opposite pattern emerged in one of the ECG datasets, with cardiac aperiodic activity steepening rather than flattening with age in a specific lower frequency range (0.25–12 Hz). This observation was particularly surprising given the flattening trend seen in other frequency ranges and datasets; it highlights how different physiological processes may drive age-related changes in aperiodic activity in a frequency-dependent and nuanced manner.

**Figure 1.**
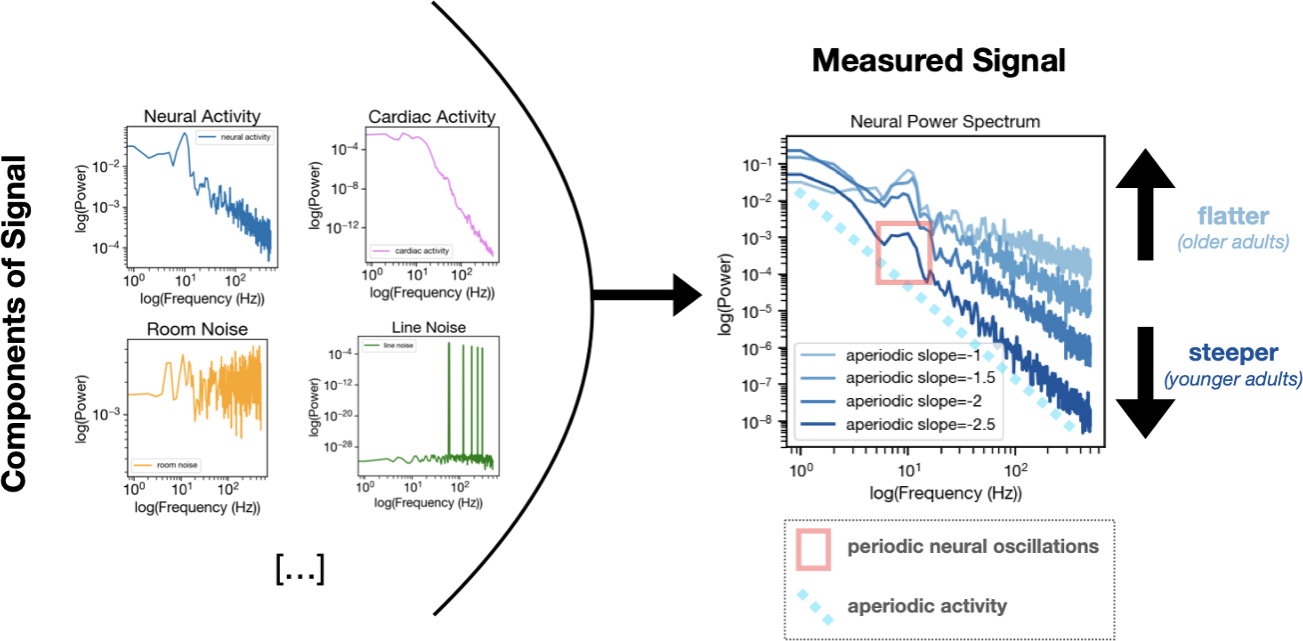
Age-related changes in aperiodic neural activity may be driven in part by aperiodic cardiac activity. Left. While often presumed to reflect purely neural sources, brain recordings are in fact composed of many independent components, which include physiological signals (both from true neural signals and from cardiac activity or other non-neural sources; top) as well as non-physiological sources of noise (such as room or line noise; bottom). Right. Calculating the power of neural activity across many frequencies results in what is known as a power spectrum, with neural activity (or ‘power’; y axis) plotted against frequency (x axis). From the power spectrum, it is possible to see both the periodic component of the signal (the wave-like element inside the red rectangle) and the aperiodic component (light blue dotted line). The slope of the aperiodic component can vary with age, being steeper in young adults and becoming flatter with age. Schmidt et al. demonstrate that the aperiodic component of a cardiac signal (not shown) can similarly ‘flatten’ with age. This finding suggests that cardiac artifacts in MEG and EEG recordings may partly contribute to the age-related neural changes previously ascribed to the brain getting older. MEG: magnetoencephalography; EEG: electroencephalography.

Age and disease are not alone in affecting aperiodic activity, as cognitive and behavioral factors can also have an influence. For instance, recordings show that neural aperiodic activity flattens when subjects perform cognitive tasks (Preston et al., 2022). Schmidt et al. detected a similar effect in the cardiac aperiodic activity of participants temporarily holding information in mind during a working memory task, suggesting that cardiac influence on neural changes may extend beyond aging.

Where do we go from here? The findings by Schmidt et al. underscore the importance of developing more sophisticated models that can accurately account for all potential sources of noise. By doing so, we can avoid being misled by our own dust; instead, we can move beyond basic observations about ‘something happening in the brain’ and towards a deeper physiological understanding of how neural circuits are changing and interacting with other age-related bodily processes.
